# The Management of the Ear, Nose and Throat in Influenza
*Communicated to the Bristol Medico-Chirurgical Society on 13th April, 1932.


**Published:** 1932

**Authors:** A. J. Wright

**Affiliations:** Lecturer on Laryngology in the University of Bristol; Surgeon in charge of the Ear, Nose and Throat Department, General Hospital, Bristol


### THE MANAGEMENT OF THE EAR, NOSE AND THROAT IN INFLUENZA.*

BY

A. J. Wright, M.B., F.R.C.S.,

*Lecturer on Laryngology in the University of Bristol; Surgeon in charge of the Ear, Nose and Throat Department, General Hospital, Bristol.*

---

Influenza being as yet an undefined complaint, I should include under the above title any acute inflammation of the pharynx and upper air passages, particularly if occurring in epidemic form. Under the management of the ear, nose and throat in such cases I propose to discuss the measures which can be most usefully employed, both to shorten the duration, and to limit the extent, of the inflammatory lesion. The simplest plan seems to be to deal first with the general constitutional side of treatment. Under this heading I should attach the greatest importance to rest in bed, a dose of calomel, and aspirin and phenacetin internally. The preparation known as S.U.P.36 has been extensively used, with the idea of cutting short the course of the infection, but I myself, at present, should wish to postpone judgment as to its value. In some of the most acute cases, however, I am convinced that an injection of scarlatinal streptococcus antitoxin (globulins), if given early and in an adequate dose of (say) 20 c.c., will

* Communicated to the Bristol Medico-Chirurgical Society on 13th April, 1932.

produce surprisingly good results. This applies particularly to cases associated with a hæmolytic streptococcal infection.

Secondly, as to the local treatment of the nose, throat and ear. It will, perhaps, clear the air if one first considers what might be done to an inflamed mucous membrane to assist resolution. In considering this, I believe it to be important to appreciate the fact that any discharge produced is the result of the inflammation and a proof of the resistance of the tissues to such inflammation. We are apt, I think, to regulate our treatment from the standpoint that it is the discharge itself that is the important factor in the disease. What can we do to an inflamed mucous membrane ? We can leave it alone (and there is a good deal to be said for this) ; we can wash it ; we can apply heat or cold, chemical antiseptics, or sera of supposed antitoxic or bactericidal properties. In dealing with a cavity with a narrow outlet we can also improve drainage by the use of vasoconstrictors, *i.e.* in the case of the nasal sinuses and middle ear, in which drainage and ventilation are of paramount importance. Dealing now with the various regions in turn :—

*The Nose and Nasal Sinuses.*—It is of importance here to teach the patient how to remove the discharge with the minimum risk of spreading the infection, particularly to the middle ear. Nose-blowing is an old - established custom, but owing to the necessity of observing social amenities it is usually performed badly. The safest method of clearing the nose is to sniff the discharge back into the nasopharynx and then to expectorate it, suction here replacing compression. If blowing is employed, only one nostril should be compressed at a time and no undue force should be used. You must all have met with cases in which the patient has associated (I believe correctly) one particular forcible blow with the onset of an acute otitis.

As far as the washing of the nasal mucosa is concerned, I believe that this is better avoided. The application of antiseptics is of doubtful utility, but the use of 5 per cent. argyrol from a spray may possibly be helpful, and at any rate does no greater harm than to stain the handkerchiefs. The use of steam inhalations with eucalyptus and menthol does perhaps do something towards reducing the swelling of the mucous membrane, thus assisting drainage. Oily solutions of menthol, chloretone, cinnamon and carbolic, from an atomizer, are probably as good as anything.

Vasoconstrictors used in solution from a spray or applied on a small piece of cotton-wool are of very great assistance, particularly in cases in which the drainage from one particular sinus is defective, as shown by the presence of pain. Of the possible vasoconstrictors adrenalin is, I believe, better avoided, as tending to be followed by an intense reaction with vaso-dilatation. Cocaine is the most useful drug, but of course it must be used with caution. If given to the patient he should not know what it is, and if from a spray nothing stronger than a 1 per cent. solution should be used. If circumstances permit of it, the application of a swab dipped in a 5 per cent. solution by the surgeon is probably a better way. In my experience cocaine can safely be employed if care be taken to avoid its entry into the stomach. As an alternative to cocaine, ephedrine in oily solution is probably the best substitute.

In cases in which there are signs of defective drainage from one or more of the sinuses, as evidenced by pain, tenderness and possibly fever, the application of heat externally by a hot bottle or fomentation is undoubtedly helpful. The adoption of a position which will tend to promote drainage from the sinus involved is also advisable, particularly after the application of a vasoconstrictor. The performance of a negative Valsalva by the patient, particularly, again, after the use of a vasoconstrictor, will sometimes uncork the obstructed orifice of a sinus. Finally, the occurrence of external swelling or the persistence of pain, tenderness and fever for more than a day or two may call for the intervention of surgery.

*The Nasopharynx.*—In many cases the complaint starts in the nasopharynx, but this region, being out of sight, tends to be out of mind. As far as local applications are concerned the region is difficult of access, and I think it is wise to be content with the use of an oily preparation from an atomizer through the nose.

*The Pharynx.*—It is curious that while acute inflammation in the nasal passages, as far as I know, never shows any involvement of the lymphatic glands, the exact converse obtains in the case of the pharynx. I think it probable that Nature here, as usual, has decided in favour of the specialist! She has handed over to the nose the function of warming, moistening and filtering the inspired air, while to the pharynx is given the duty of dealing with infections, by handing them on into the lymphatic circulation and thus calling up the general resistance of the body. The use of antiseptic washes in the case of the pharynx is not open to the same objections as obtain in the case of the nose. The mucous membrane here is a much more robust structure than the ciliated epithelium of the nose, and, in addition, it is used to being bathed in liquids. The time-honoured method of gargling, when it can be carried out, is effective, and aspirin in suspension relieves the pain more than anything else. In the more acute cases a better proceeding is for the patient to syringe a jet of fluid into the back of the pharynx through the mouth with the head bent forwards. The fluid used should be hot and should contain bicarbonate of soda to dissolve the stringy mucus which is so distressing. It is customary to add some mild antiseptic, and I think that this is harmless. I myself am not in favour of the use of paints to the pharynx in acute inflammatory lesions, but if such are used they should be applied gently and be non-irritating. The customary Mandl's paint (a solution of iodine in glycerine) is, I am sure, frequently harmful. The associated swelling and tenderness of the cervical glands call for an external application, and from the patient's point of view I think there is nothing better than antiphlogistine.

*The Larynx.*—As this is the most overworked organ in the body (in most cases, at any rate), the great difficulty is to obtain rest to the inflamed parts. Bed, a taciturn attendant, and an exciting novel are helpful. In addition, if there is much cough, heroin is strongly indicated. As in the case of the nose, steam inhalations are customary and are possibly helpful.

*The Ear.*—Cases with middle-ear involvement form one of the most important classes with which we have to deal. The avoidance of forcible nose-blowing, nasal washes and other insults to the nose will often prevent the involvement of the middle ear. Should such occur, it is important to realize that an acute middle-ear inflammation may proceed to any stage and then resolve. Although the stages merge into one another, one can recognize the following: hyperæmia of the lining of the middle ear; a serous exudate into the middle ear, or finally a purulent exudate into the middle ear. A similar state of affairs obtains in the mastoid antrum and cells, but the conditions in the mastoid cavities and the middle ear do not necessarily run a parallel course. It should be realized that the occurrence of an acute middle-ear inflammation is accompanied by swelling of the lining of the Eustachian tube, the obstruction so produced making the middle ear a closed cavity.

The variable course taken by cases of middle-ear inflammation renders their treatment very difficult, as the experience of one case is a fallacious guide to the next. Pain, fever and some defect in hearing are almost invariable indications of an acute otitis. As long as the membrane is intact it is not possible to introduce remedies into the middle ear, but it is customary in the early stages to drop something into the meatus. Opium and its preparations are frequently used as a local application, but so employed are, I believe, useless; on the other hand, the majority of cases of acute otitis have so much pain that they are the better for opium internally. Solutions of carbolic and glycerine are also frequently used, but the tendency is to use them much too strong, with resulting dermatitis of the meatus. I myself now depend on pure glycerine, but should carbolic be added it should not be in a greater strength than 5 per cent. Another fashionable drug, mercurochrome, is sometimes employed at this stage, but I know of no preparation which is more irritating to the surgeon. It can do no good through the intact membrane, but by dyeing everything red it prevents any possibility of watching the progress of the disease! Heat to the side of the head is usually comforting and seems rational.

Finally, in many cases of acute otitis the question of surgery arises. When should a membrane be incised? There are two schools of thought in regard to this question: those who consider that every membrane which shows any signs of inflammation should be incised, and those who consider that this little operation should be reserved for cases in which there are definite indications. I belong to the latter group. The presence of a bulging membrane, with fever that has persisted for more than forty-eight hours, are, in my opinion, such indications. Apart from the question of incision, one must consider the still more difficult question of when a mastoid process should be opened and drained. Here again my outlook is somewhat conservative. With the exception of what may be described as fulminating cases, in which high fever, acute toxæmia and widespread mastoid tenderness are present from the onset of the complaint, I believe that it is better not to open and drain the mastoid process in the earliest stages of the inflammation. If one waits for a few days both local and general resistance to the infection are developed, and it will be found that the post-operative course of the illness is much less stormy than if one operates at a very early stage.

Finally, what should be the routine care of an acute middle-ear suppuration in which discharge is established whether by nature or by art? My practice is to continue the use of sterile glycerine drops for a few days, keeping the ear covered with a sterile dressing and preceding the putting in of the drops by a careful wiping out of the meatus under as sterile conditions as possible. As soon as the temperature has fallen and the pain has ceased I believe in following as dry a line of treatment as is possible. The meatus should be mopped dry frequently, should be left uncovered at the earliest opportunity, and boracic or some other antiseptic powder should be insufflated to prevent decomposition of the discharge with resulting mixed infection.

